# Antidiabetic Effect and Mode of Action of Cytopiloyne

**DOI:** 10.1155/2013/685642

**Published:** 2013-03-13

**Authors:** Cicero Lee-Tian Chang, Hsien-Yueh Liu, Tien-Fen Kuo, Yi-Jou Hsu, Ming-Yi Shen, Chien-Yuan Pan, Wen-Chin Yang

**Affiliations:** ^1^Department of Veterinary Medicine, National Chung Hsing University, 402 Taichung, Taiwan; ^2^Agricultural Biotechnology Research Center, Academia Sinica, 128 Academia Road, Section 2, Nankang, 115 Taipei, Taiwan; ^3^Research Center, China Medical University and Hospital, Graduate Institute of Clinical Medical Sciences, China Medical University, 40402 Taichung, Taiwan; ^4^Institute of Zoology, National Taiwan University, 10617 Taipei, Taiwan; ^5^Department of Life Sciences, National Chung Hsing University, 402 Taichung, Taiwan

## Abstract

Cytopiloyne was identified as a novel polyacetylenic compound. However, its antidiabetic properties are poorly understood. The aim of the present study was to investigate the anti-diabetic effect and mode of action of cytopiloyne on type 2 diabetes (T2D). We first evaluated the therapeutic effect of cytopiloyne on T2D in db/db mice. We found that one dose of cytopiloyne reduced postprandial glucose levels while increasing blood insulin levels. Accordingly, long-term treatment with cytopiloyne reduced postprandial blood glucose levels, increased blood insulin, improved glucose tolerance, suppressed the level of glycosylated hemoglobin A_1c_ (HbA_1c_), and protected pancreatic islets in db/db mice. Next, we studied the anti-diabetic mechanism of action of cytopiloyne. We showed that cytopiloyne failed to decrease blood glucose in streptozocin- (STZ-)treated mice whose **β** cells were already destroyed. Additionally, cytopiloyne dose dependently increased insulin secretion and expression in **β** cells. The increase of insulin secretion/expression of cytopiloyne was regulated by protein kinase C**α** (PKC**α**) and its activators, calcium, and diacylglycerol (DAG). Overall, our data suggest that cytopiloyne treats T2D via regulation of insulin production involving the calcium/DAG/PKC**α** cascade in **β** cells. These data thus identify the molecular mechanism of action of cytopiloyne and prove its therapeutic potential in T2D.

## 1. Introduction

Insulin is indispensable for glucose homeostasis in mammals. Insulin biosynthesis at transcriptional and translational levels, and its secretion in *β* cells, is well regulated by blood glucose [1]. Calcium ions, potassium ions, phospholipase C, DAG, phosphatidylinositol triphosphate (IP3), PKC, and protein kinase A (PKA) are involved in insulin secretion and, likely, insulin biosynthesis in pancreatic *β* cells [2]. On binding to insulin, an insulin receptor initiates a signaling cascade and eventually causes glucose uptake in peripheral tissues. Any defect in insulin synthesis/secretion or action, or both, may result in hyperglycemia, a major pathological feature of type 2 diabetes (T2D) [3]. Such hyperglycemia is detrimental to *β* cells and insulin target tissues, and this glucotoxicity is clinically relevant as a cause of diabetes-related complications such as nephropathy, retinal blindness, neuropathy, peripheral gangrene, and cardiovascular disease [4]. Therefore, maintenance of glycemic homeostasis is the most common therapeutic aim for patients with T2D.

Diabetes is a life-threatening metabolic disease, afflicting around 5% of the world population. Over 90% of the diabetic population is diagnosed with T2D mellitus [5, 6]. Current antihyperglycemic drugs are insulin secretagogues, insulin sensitizers, inhibitors of sugar cleavage, and glucagon-like peptide-1 (GLP-1), all of which control homeostasis of blood sugar by different mechanisms [7]. Common drawbacks of such drugs include significant side effects, decreased efficacy over time, low cost-effectiveness, and only partial anti-diabetic effect of each individual drug [8]. Of note, secretagogues with the ability to prevent adverse effects (e.g., weight gain and hypoglycemia), to stimulate insulin biosynthesis, or to protect *β* cells from death are rare [7, 9]. GLP-1, an injectable peptide drug, may be the only one reported to fit these criteria [10]. In view of patients' welfare, there is still an obvious need for development of antidiabetics that protect against hypoglycemia, enhance insulin synthesis, or improve *β*-cell protection.

Plants are an extraordinary resource for anti-diabetic remedies [11, 12]. One outstanding example is metformin, a derivative of guanidine that was first isolated from French lilac and is a commonly prescribed insulin sensitizer for treatment of T2D [13]. Further, extracts prepared from the plant *Bidens pilosa*, a member of the Asteraceae family, were shown to have anti-diabetic properties in alloxan-treated mice [14] and have been used to treat patients with diabetes in America, Africa, and Asia [11, 15]. Two polyacetylenes isolated from *B. pilosa* have demonstrated anti-diabetic properties by two different laboratories [16, 17]. More recently, another polyacetylene, cytopiloyne, was identified in *B. pilosa* and shown to be highly potent in the prevention of type 1 diabetes via T-cell regulation [18]. *B. pilosa* and its three polyacetylenes showed glucose-lowering activities in diabetic mice [16, 19, 20]. However, the long-term therapy and mechanism of these three polyacetylenes for T2D are not known.

The db/db mice whose leptin receptor gene is mutated spontaneously develop diabetes because of insulin resistance [21]. STZ-treated mice represent a chemical-inducible model that exhibits insufficient insulin production [22]. Both models reflect main causes of T2D [23]. In this study, we studied the anti-diabetic potential and mechanism of cytopiloyne in db/db mice and STZ-treated C57BL mice and in pancreatic *β* cells.

## 2. Materials and Methods

### 2.1. Ethics Statement

All animals were maintained and handled according to the institutional guidelines and the protocol was approved by the Academia Sinica Animal Care and Utilization Committee (protocol number: OMiIBAYW2010043).

### 2.2. Chemicals, Cells, and Animals

Dimethyl sulfoxide (DMSO), STZ, nimodipine, EGTA, metformin, glimepiride, brefeldin A, hematoxylin, eosin, phorbol 12-myristate 13-acetate (PMA), glimepiride, 1-stearoyl-2-arachidonoyl-*sn*-glycerol, cholesterol, and diaminobenzidine tetrahydrochloride were purchased from Sigma-Aldrich (MO, USA). Antiactin, anti-HA, and anti-insulin antibodies were purchased from Santa Cruz Biotechnology (CA, USA). Insulin (Novo Nordisk, NJ, USA) and anti-PKC*α* (Abcam, MA, USA) and antiphospho-PKC*α* (Millipore, MA, USA) antibodies were purchased. Cytopiloyne was prepared to 98% purity from *B. pilosa* as previously described [17, 18]. Cytopiloyne dissolved in DMSO was stored in a light protected vial at −20°C. After one year storage, more than 90% of the isolated cytopiloyne was stable, as was confirmed by structural determination by NMR spectroscopy. RIN-m5F cells (CRL-11605), a rat *β*-cell line, were obtained from the American Type Culture Collection. Primary pancreatic islets were isolated from fasted Wistar rats obtained from the National Laboratory Animal Center (NLAC) in Taipei, Taiwan. C57BL and db/db mice [24] were obtained from the NLAC. All animals were maintained in the institutional animal facility and handled according to the guidelines of the Academia Sinica Institutional Animal Care and Utilization Committee.

### 2.3. Drug Administration in db/db Mice

For single-dose administration, diabetic db/db males aged 6 to 8 weeks, with free access to food, were grouped and tube fed with either 0.2 mL vehicle (1 *μ*L DMSO per 1 mL of PBS), cytopiloyne (0.1, 0.5, and 2.5 mg/kg body weight (BW)), or glimepiride (2.5 mg/kg BW). After 0.5 h, the levels of postprandial blood sugar and insulin from the mice were monitored for an additional 4 h. For continuous administration, diabetic db/db males were grouped and tube-fed with vehicle, cytopiloyne (0.5 or 2.5 mg/kg/day), or glimepiride (2.5 mg/kg/day) for the indicated time, while levels of blood sugar, insulin, and glycosylated HbA_1c_ and glucose tolerance in these mice were determined. Unless otherwise indicated, the mice were fasted for 16 h and then postprandial blood glucose and insulin in these mice were measured. A portion of mice were sacrificed for immunohistochemical staining. The rest were maintained to determine survival rates.

### 2.4. Drug Administration in STZ-Treated Mice

To deplete pancreatic *β* cells in mice, 6-week-old C57BL females were intraperitoneally injected with STZ at 200 mg/kg. STZ-treated females with postprandial blood sugar over 500 mg/dL were grouped. Each group was either tube-fed with vehicle (1 *μ*L DMSO per 1 mL of PBS), cytopiloyne (0.1, 0.5, and 2.5 mg/kg), or glimepiride (2.5 mg/kg) or injected with insulin at 2.5 IU/kg BW. Blood glucose levels were monitored for 4 h. To distinguish sensitizer activity from releaser activity of cytopiloyne, STZ-treated C57BL mice were tube-fed with vehicle, glimepiride (2.5 mg/kg), metformin (60 mg/kg), and cytopiloyne (0.5 and 2.5 mg/kg) 1 h before insulin injection (2.5 IU/kg). Blood glucose levels in the mice were monitored from 0 to 4 h after insulin injection.

### 2.5. Measurement of Glucose, Insulin, and HbA_**1c**_

Glucose levels in mouse blood samples were measured using an Elite glucometer (Bayer, PA, USA). Insulin levels in blood samples or islet cell supernatants were determined by ELISA assays (Mercodia, Uppsala, Sweden). The levels of glycosylated HbA_1c_ in blood samples were measured using a DCA 2000 analyzer (Bayer, PA, USA).

### 2.6. Insulin Secretion

 Pancreatic islets (10 islets/mL) from fasted male Wistar rats were incubated with Krebs-Ringer bicarbonate (KRB) buffer [25] containing vehicle (1 *μ*L DMSO per mL KRB buffer), glimepiride, or cytopiloyne in the absence or presence of glucose for 30 min. The KRB buffer was then collected for ELISA assays.

### 2.7. Intraperitoneal Glucose Tolerance Test (IPGTT)

Male db/db mice were administered either vehicle, cytopiloyne at 0.5 and 2.5 mg/kg/day, or glimepiride at 2.5 mg/kg/day for the indicated time. The mice were fasted for 16 h before the glucose tolerance test. On days 0 and 42, each group received an oral dose (0.2 mL) of vehicle (1 *μ*L DMSO per 1 mL of PBS), glimepiride, or cytopiloyne (time 0) and one intraperitoneal injection with glucose (0.5 g/kg) 0.5 h later. The levels of blood sugar were monitored from −0.5 to 3 h after glucose administration.

### 2.8. Immunohistochemistry

 Pancreata from db/db males with continuous drug administration were snap frozen in *OCT* compound and stained with hematoxylin and eosin or anti-insulin antibody, followed by diaminobenzidine tetrahydrochloride development as previously published [18]. Optimal cutting temperature compound (OCT) is an inert cryosection medium. Multiple parallel sections of each pancreas were analyzed by light microscopy.

### 2.9. Intracellular Staining for Insulin

Rat pancreatic islets were incubated with vehicle alone (1 *μ*L DMSO per 1 mL of PBS), cytopiloyne, or glimepiride for 24 h. The islet cells were dissociated into single-cell suspension, stained with anti-insulin antibody and analyzed by fluorescence-activated cell sorting (FACS).

### 2.10. Real-Time RT-PCR Analysis

Rat pancreatic islets were incubated with vehicle (1 *μ*L DMSO per 1 mL of PBS), cytopiloyne, or glimepiride for 24 h. Total RNA isolated from these islets was extracted and converted to cDNA. Real-time RT-PCR was performed with the above cDNA using insulin primers (5′-TGCGGGTCCTCCACTTCAC-3′ and 5′-GCCCTGCTCGTCCTCTGG-3′) or L13 primers (5′-AGA TAC CAC ACC AAG GTC CG-3′ and 5′-GGA GCA GAA GGC TTC CTG-3′).

### 2.11. Transfection and Luciferase Assays

The phINS-Luc and pRL-TK plasmids contain the human insulin promoter (2347 bp) from the phINS-DCR3 vector [26] linked to the firefly luciferase gene and the thymidine kinase promoter linked to the *Renilla *luciferase reporter gene, respectively. RIN-m5F cells were transfected with pHACE-PKC*α* DN plasmid (a gift from Dr J.-W. Soh), pcDNA3 (a control plasmid), phINS-Luc, and/or pRL-TK by lipofectamine or electroporation. After a 24 h recovery, the cells were treated with vehicle (1 *μ*L DMSO per 1 mL of medium), glucose, GF109203X (a PKC inhibitor), glimepiride, or cytopiloyne for an additional 24 h. Subsequently, dual luciferase assays were performed as described [18].

### 2.12. Detection of Intracellular Calcium

RIN-m5F cells were preloaded with Fura 2-AM (5 *μ*M) in modified Krebs-Henseleit buffer for 30 min at 25°C for 1 h. After washing, the cells were stimulated with 16.7 mM glucose or cytopiloyne (7, 14, and 28 *μ*M). Intracellular calcium was measured using a fluorescence spectrophotometer (CAF 110, Jasco, Tokyo, Japan) at the excitation wavelengths of 340 and 380 nm and emission wavelength of 500 nm. The ratio of fluorescence intensity at 340 nm to that at 380 nm represents the level of intracellular calcium.

### 2.13. Extraction and Measurement of DAG

RIN-m5F cells were treated with glucose, cytopiloyne, PMA, and glimepiride for 5 min. Total lipids were extracted with ethyl acetate as previously described [27]. The cells were separated on a silica thin layer plate with the first developer of ethyl acetate : acetic acid : trimethylpentane (9 : 2 : 5) and the second developer of hexane : diethylether : methanol : acetic acid (90 : 20 : 3 : 2). The spot of DAG and cholesterol in each sample was visualized with 15% sulfuric acid and identified by 1-stearoyl-2-arachidonoyl-*sn*-glycerol and cholesterol.

### 2.14. Western Blot

**Figure 1:**
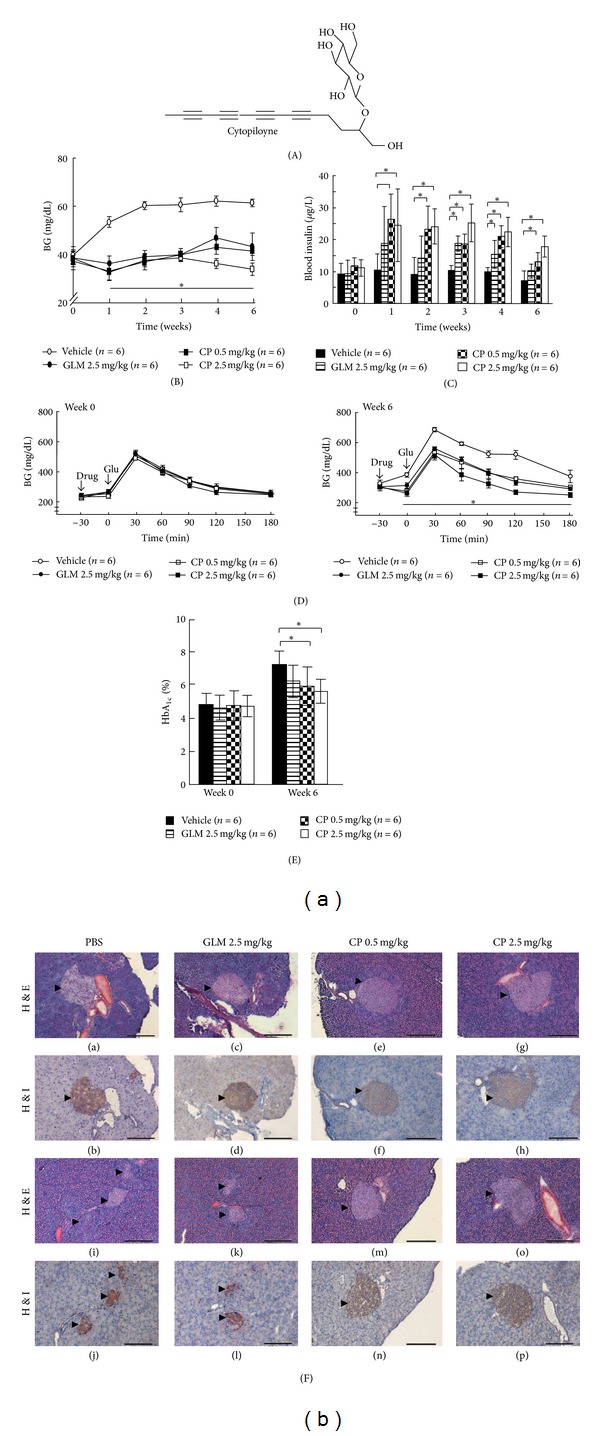
Anti-diabetic effects of cytopiloyne in db/db mice during long-term treatment. (A) Chemical structure of cytopiloyne. (B) Four groups of 6 to 8-week-old diabetic db/db mice were tube-fed with vehicle, cytopiloyne (CP, 0.5 and 2.5 mg/kg/day), or glimepiride (GLM, 2.5 mg/kg/day) from 0 to 6 weeks. Postprandial blood glucose (BG) levels in these mice were measured. (C) Blood insulin levels from the above mice (B). (D) IPGTT was performed in the above db/db mice (B) on weeks 0 and 6 after-treatment, and blood glucose levels were monitored for 3.5 h. (E) The percentage of glycosylated HbA_1c_ in whole blood from the above mice (B) was determined 0 and 6 weeks after-treatment. (F) Pancreata of 8- and 16-week-old db/db males, which had received the same treatment as described in (B) for 2 (images a–h) and 10 (images i–p) weeks, were stained with hematoxylin and eosin (H&E, images (a), (c), (e), (g), (i), (k), (m), and (o)) or hematoxylin and an antibody against insulin (H&I, images (b), (d), (f), (h), (j), (l), (n), and (p)). Arrowheads indicate pancreatic islets. Scale bars, 200 *μ*m. Results are expressed as mean ± SEM from 3 independent experiments, and *P* (*) < 0.05 was considered to be statistically significant. The number of mice (*n*) is indicated in parentheses.

RIN-m5F cells were starved in KRB buffer for 30 min. The cells were then treated with vehicle, PMA, 16.7 mM glucose, and cytopiloyne in the absence or presence of EGTA and nimodipine for the indicated time. After extensive washing, the cells were pelleted. The total lysate, cytosolic fraction, and membrane fraction were prepared, followed by SDS-polyacrylamide gel electrophoresis. The membrane was blotted with anti-PKC*α*, anti-phospho-PKC*α*, and anti-actin antibodies. The expression level of HA-tagged PKC*α*-DN in RIN-m5F cells was confirmed using Western blot with anti-HA antibody. The effect of GF109203X on PKC*α* inactivation in *β* cells was confirmed by Western blot (see the species list in the Supplementary Material of Figure  1 available online at http://dx.doi.org/10.1155/2013/685642). 

### 2.15. Statistical Analysis

Data from three independent experiments or more are presented as mean ± SEM. ANOVA was used for statistical analysis of differences between groups, and *P* (*) less than 0.05 was considered to be statistically significant.

## 3. Results

### 3.1. Beneficial Effect of Cytopiloyne on Glucose Lowering, Glucose Tolerance Test, Glycosylation of HbA_**1c**_, and Islet Preservation

**Figure 2:**
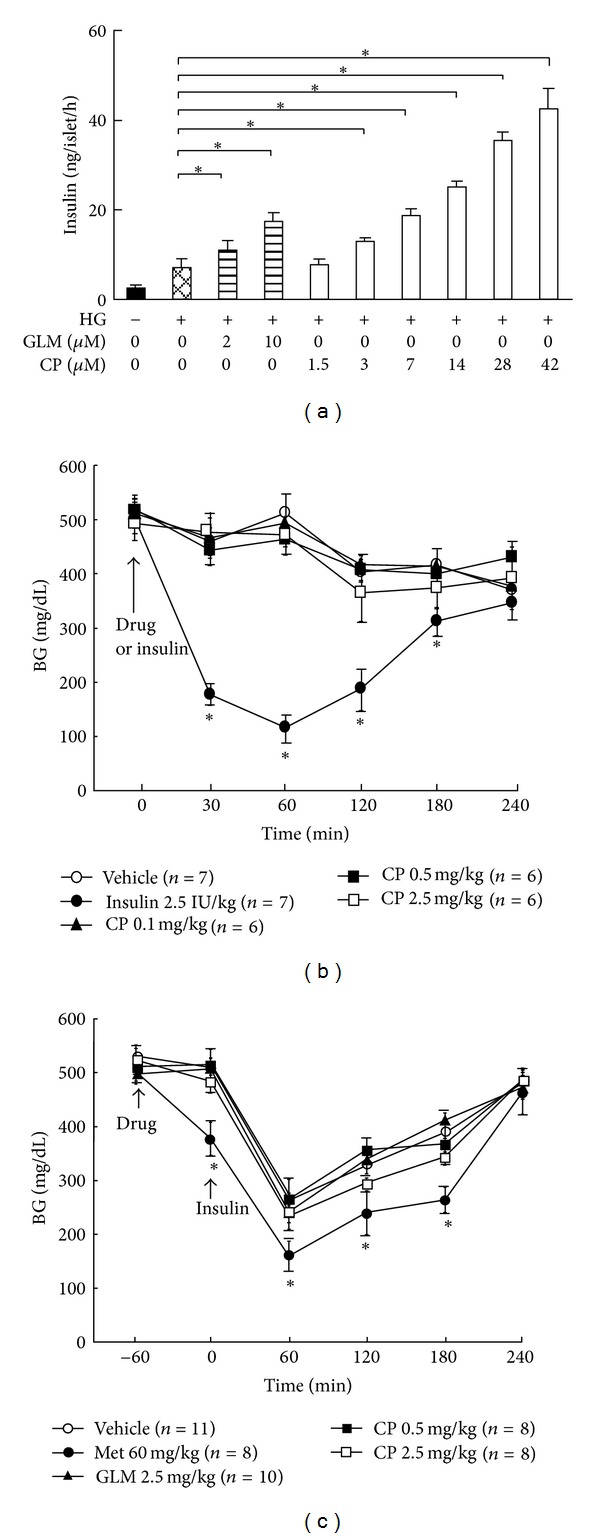
Cytopiloyne-mediated insulin secretion depends on pancreatic *β* cells. (a) Rat pancreatic islets were incubated with KRB buffer containing vehicle, glimepiride (GLM, 0 to 10 *μ*M), or cytopiloyne (CP, 1.5 to 42 *μ*M) in the absence or presence of 16.7 mM glucose (HG). The insulin levels were determined using an insulin ELISA kit. The data are presented as mean ± SEM of 3 independent experiments. (b) Fed C57BL mice, which had already received an injection of STZ, were administered an oral dose of vehicle, cytopiloyne (CP, 0.1, 0.5, and 2.5 mg/kg), and an intraperitoneal injection of insulin (Ins, 2.5 IU/kg). Postprandial blood sugar levels in the STZ-treated mice were determined using a glucometer. (c) Fed C57BL mice, which had already received STZ, were orally administered a single dose of vehicle, cytopiloyne (CP, 0.5 and 2.5 mg/kg), glimepiride (GLM, 2.5 mg/kg), or metformin (Met, 60 mg/kg), followed by an intraperitoneal injection with insulin (Ins). Postprandial blood sugar levels in the STZ-treated mice were determined using a glucometer. Results are expressed as mean ± SEM from 3 independent experiments, and *P* < 0.05 was considered to be statistically significant (*). The number of mice (*n*) is indicated in parentheses.

We and others have previously identified three polyacetylenes present in* B. pilosa* that exhibit antihyperglycemic activities in different diabetic models [16–20]. However, their long-term benefit and mode of action remained unclear. In the study, we investigated the therapeutic effect and mechanism of cytopiloyne, a polyacetylenic glucoside (Figure 1(A)), on T2D. Glimepiride, an anti-diabetic sulfonylurea drug, acts to enhance insulin secretion in pancreatic *β* cells and, in turn, reduces blood glucose. We first evaluated single-dose effects of cytopiloyne on diabetic db/db mice. We found that like glimepiride (2.5 mg/kg), cytopiloyne at doses of 0.1, 0.5, and 2.5 mg/kg significantly reduced postprandial blood glucose levels in a dose-dependent manner in diabetic db/db mice (Table 1). We also compared blood insulin levels in the same mice. Both glimepiride and cytopiloyne significantly elevated the blood insulin levels in db/db mice compared to vehicle alone (Table 2). These data showed that a single dose of cytopiloyne had anti-hyperglycemic and insulin-releasing effects on db/db mice. Next, we investigated long-term therapeutic effects of cytopiloyne in diabetic db/db mice. We found that 0.5 mg/kg cytopiloyne had similar blood sugar-lowering effects on fed db/db mice as glimepiride at 2.5 mg/kg (Figure 1(B)). Additionally, cytopiloyne was slightly more efficacious at 2.5 mg/kg than glimepiride at 2.5 mg/kg. Consistently, cytopiloyne increased blood insulin levels to a greater extent than glimepiride and this increase was dose dependent (Figure 1(C)). We also evaluated the effect of cytopiloyne on glucose tolerance. IPGTT assays showed no difference in glucose tolerance in treated and control mice at week 0 (upper panel, Figure 1(D)). By contrast, cytopiloyne treatment for 6 weeks improved glucose tolerance in db/db mice to a greater extent than glimepiride at the same dose (2.5 mg/kg) (lower panel, Figure 1(D)). Glycosylated HbA_1c_ is known to be an excellent indicator of long-term glycemic control. Therefore, we examined the percentage of glycosylated HbA_1c_ in db/db mice following different treatments. In the blood from 6- to 8-week-old db/db mice, 4.8% HbA_1c_ was glycosylated. However, by 12 to 14 weeks of age, this value had risen to 7.3% in untreated db/db mice. By contrast, 6.3%, 6%, and 5.6% of HbA_1c_ were glycosylated in the blood of age-matched mice following treatment with 2.5 mg/kg glimepiride or with 0.5 mg/kg or 2.5 mg/kg cytopiloyne, respectively (Figure 1(E)). These data suggest that cytopiloyne, which reduced glycosylated HbA_1c_ by 1.3% and 1.7% at concentrations of 0.5 mg/kg or 2.5 mg/kg, respectively, achieves relatively tighter glycemic control than glimepiride, which only decreased glycosylated HbA_1c_ by 1%, in db/db mice. Diabetic db/db mice usually develop severe atrophy of pancreatic islets. We assessed the protective effect of cytopiloyne on islet destruction in db/db mice aged 8 and 16 weeks, which corresponded to early and chronic stages of diabetes, respectively [28]. There was no significant difference in pancreatic islets of treated and untreated db/db mice at 6 to 8 weeks of age. Twelve- to 14-week-old db/db mice, which had received a long-term treatment with vehicle control and glimepiride, had sporadic islets. In sharp contrast, the age-matched db/db mice with cytopiloyne treatment showed much greater preservation of islet structure (Figure 1(F)). Accordingly, cytopiloyne treatment resulted in a better survival rate as compared to treatment with glimepiride or vehicle in db/db mice (see the species list in the Supplementary Material of Table  1 available online at http://dx.doi.org/10.1155/2013/685642). We also confirmed the preventive effect of cytopiloyne on T2D in db/db mice aged 4 weeks that has been previously reported [29, 30]. Cytopiloyne failed to stop the development of T2D in db/db mice, but it significantly reduced hyperglycemia in these mice compared to the control cohort (see the species list in the Supplementary Material of Figure  2 available online at http://dx.doi.org/10.1155/2013/685642). It should be noted that the blood glucose levels of db/db mice in preventive experiments (see the species list in the Supplementary Material of Figure  2(a) available online at http://dx.doi.org/10.1155/2013/685642) and therapeutic experiments (Figure 1(B)) were dissimilar because the ages of the mice examined were different. Collectively, cytopiloyne treatment for diabetes was better than glimepiride in terms of both dosage and therapeutic effects.

### 3.2. Cytopiloyne Acts as an Insulin Secretagogue rather than a Sensitizer

**Figure 3:**
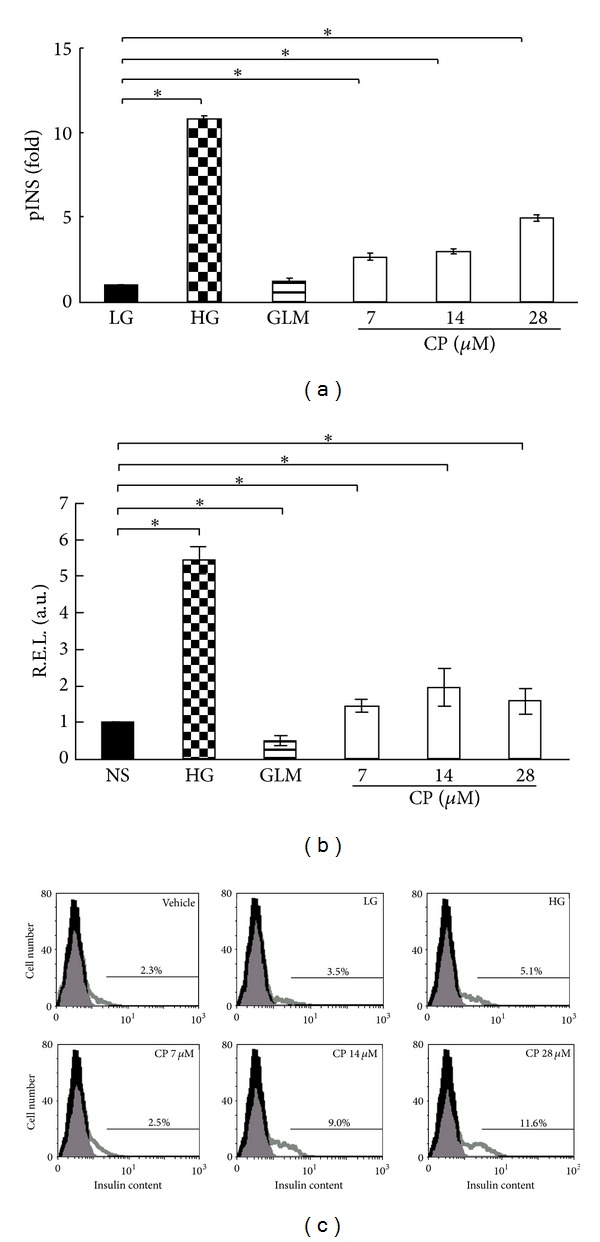
Increase in insulin mRNA and protein content by cytopiloyne in pancreatic islets. (a) RIN-m5F *β* cells transfected with phINS-Luc and pRL-TK plasmids were incubated with medium containing 3.3 mM glucose in the presence of vehicle (LG), glimepiride (GLM, 10 *μ*M), and cytopiloyne (7, 14, or 28 *μ*M) or 16.7 mM glucose (HG). Insulin promoter activity expressed as fold change relative to vehicle-treated control was measured using dual luciferase assays. (b) The relative expression level (R.E.L.) of insulin relative to L13 in rat primary pancreatic islets, which were already treated with 3.3 mM glucose in the presence of vehicle (LG), glimepiride (GLM, 10 *μ*M), or cytopiloyne (7, 14, or 28 *μ*M) or 16.7 mM glucose (HG) for 24 h, was determined by real-time RT-PCR. (c) Rat pancreatic islets received the same treatments as the islets in (b) in the presence of brefeldin A for 24 h. After anti-insulin antibody staining, these cells underwent FACS analysis. The percentage of insulin-positive *β* cells is shown. Results are expressed as mean ± SEM from 3 independent experiments, and *P* < 0.05 was considered to be statistically significant (*).

The sugar-reducing and insulin-increasing effects of cytopiloyne raised the possibility that cytopiloyne controls blood sugar in db/db mice primarily through stimulating insulin production from *β* cells. Rat primary pancreatic islets are commonly used to test insulin secretion/synthesis because rats have more abundant pancreatic islets than mice and the islets of both species respond to glucose similarly [1]. To examine the role of cytopiloyne in insulin secretion, we treated rat islets with cytopiloyne in KRB buffer containing 16.7 mM glucose. We found that cytopiloyne effectively enhanced insulin secretion in high glucose medium (Figure 2(a)) as well as glucose-free and low-glucose media (see the species list in the Supplementary Material of Figure  3 available online at http://dx.doi.org/10.1155/2013/685642). To confirm that cytopiloyne reduced hyperglycemia by stimulating insulin production from pancreatic *β* cells *in vivo*, we tested its ability to reduce hyperglycemia and to augment insulin levels in STZ-treated C57BL mice whose *β* cells were already depleted. As expected, cytopiloyne lost its ability to regulate both responses in these mice (Figure 2(b)). In sharp contrast, insulin treatment still diminished blood glucose levels in *β*-cell-depleted mice (Figure 2(b)). To exclude the possibility that cytopiloyne is an insulin sensitizer, we administrated STZ-treated C57BL/6 mice with an oral dose of vehicle, glimepiride, metformin, or cytopiloyne 60 min before an insulin injection. Both cytopiloyne and glimepiride had little, if any, lowering effect on blood sugar in these mice. However, metformin, an anti-diabetic biguanide drug, acts to sensitize insulin signaling and, in turn, significantly reduced blood glucose levels compared to vehicle alone in these mice (Figure 2(c)). Overall, our results support an insulin-releasing role of cytopiloyne in *β* cells. 

### 3.3. Cytopiloyne Elevates the Level of Insulin mRNA and Protein in Pancreatic Islets

Glucose is known to modulate transcription, translation, and secretion of insulin in pancreatic *β* cells [1]. However, current secretagogues act to increase insulin secretion but not synthesis. We have shown that cytopiloyne increases insulin secretion from rat islets (Figure 2(a)). Therefore, we also evaluated the effect of cytopiloyne on insulin expression. We first used an insulin promoter-driven reporter construct to test the effect of different treatments on insulin transcription. Glimepiride had no significant effect on insulin transcription in RIN-m5F *β* cells, a rat *β*-cell line, compared to the low-glucose control (3.3 mM). By contrast, high glucose (16.7 mM) upregulated insulin transcription eleven times; 28 *μ*M cytopiloyne augmented insulin transcription five times, and this increase was dose dependent (Figure 3(a)). Next, we examined the expression levels of insulin mRNA relative to those of L13, a house-keeping control gene, in rat islet cells pretreated with low glucose, high glucose, 10 *μ*M glimepiride, or cytopiloyne at 7, 14, and 28 *μ*M for 24 h. Glimepiride slightly decreased insulin transcription. By contrast, a high concentration of glucose up-regulated insulin transcription five times, while 28 *μ*M cytopiloyne resulted in doubled insulin transcription (Figure 3(b)). Further, we examined the effect of cytopiloyne on insulin content inside pancreatic islet cells. FACS is a sensitive method to detect levels of an intracellular protein at the level of an individual cell. Therefore, we used FACS to monitor the content of intracellular insulin. Glucose treatment (16.7 mM) increased the intracellular insulin levels in these cells from 2.3% to 5.1% (Figure 3(c)). Consistent with the effect of cytopiloyne on insulin transcription, 28 *μ*M cytopiloyne increased the intracellular insulin levels 5-fold compared to control treatment in these cells, and this effect on insulin content was dose dependent (Figure 3(c)). The overall data suggest that cytopiloyne stimulates insulin expression in pancreatic *β* cells. 

### 3.4. Cytopiloyne Increases Calcium Influx, DAG Generation, and PKC*α* Activation

**Figure 4:**
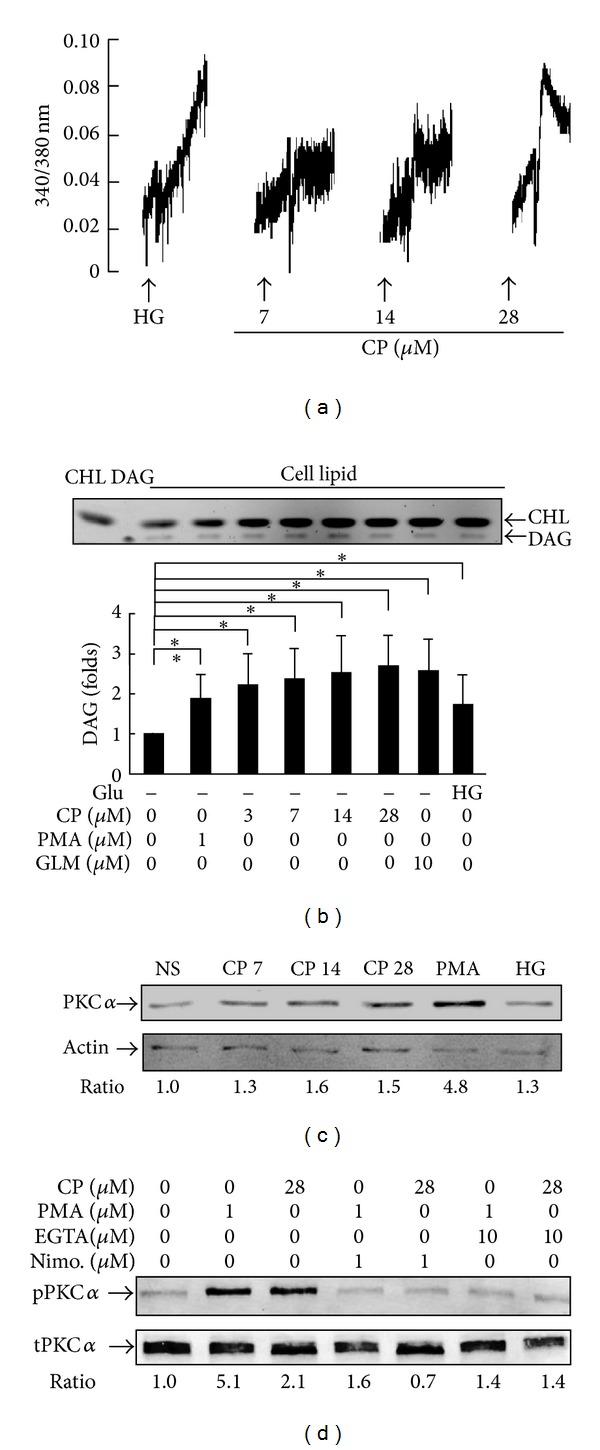
Effects of cytopiloyne on calcium mobilization, DAG generation, and PKC*α* activation. (a) After Fura 2-AM loading, RIN-m5F cells were stimulated with 16.7 mM glucose (HG) and cytopiloyne (CP) at 7, 14, and 28 *μ*M. The level of intracellular calcium, as shown by the 340/380 nm ratio, was detected using a fluorescence spectrophotometer. (b) RIN-m5F cells were stimulated with glucose, cytopiloyne (CP), PMA, and glimepiride (GLM). Total cell lipids and their commercial standards, DAG and cholesterol (CHL), were resolved on a silica thin layer plate. The quantity of DAG and cholesterol in each sample is replotted into histograms. (c) RIN-m5F cells were incubated with vehicle (NS), cytopiloyne (CP, 7, 14, and 28 *μ*M), PMA (1 *μ*M), and 16.7 mM glucose (HG). Membrane proteins of each sample were subjected to Western blot with anti-PKC*α* and anti-actin antibodies. (d) RIN-m5F cells were incubated with vehicle, cytopiloyne (28 *μ*M), and PMA (1 *μ*M) in the absence or presence of EGTA (10 *μ*M) and nimodipine (Nimo, 1 *μ*M). Total proteins were subjected to Western blot with anti-PKC*α* (t-PKC*α*) and anti-phospho-PKC*α* (p-PKC*α*) antibodies.

Secondary messengers such as calcium and DAG are involved in a variety of signaling pathways in *β* cells [31–35]. We wanted to understand the mechanism of cytopiloyne in the insulin expression and the release in *β* cells. Our data showed that RIN-m5F cells responded to glucose and cytopiloyne in a similar way to primary rat islet cells (Figures 3(a) and 3(b)). Therefore, we used RIN-m5F cells to test the effect of cytopiloyne on calcium mobilization. We found that 16.7 mM glucose significantly increased intracellular calcium in *β* cells (Figure 4(a)). Similarly, cytopiloyne increased the level of intracellular calcium in a dose-dependent manner (Figure 4(a)). Next, we determined the effect of cytopiloyne on the production of lipids, DAG, and cholesterol, in RIN-m5F cells. PMA, glimepiride, and 16.7 mM glucose significantly increased the level of DAG in comparison with the vehicle control (Figure 4(b)). Of note, cytopiloyne dose dependently increased the level of DAG but not cholesterol (Figure 4(b)). Because PKC*α* has been previously implicated in insulin secretion of *β* cells [31, 33], we next assessed the effect of cytopiloyne on PKC*α* activation by examining its translocation and phosphorylation. Like PMA and 16.7 mM glucose, cytopiloyne dose dependently increased the membrane portion of PKC*α* (Figure 4(c)). Besides, like PMA, cytopiloyne increased the phosphorylation of PKC*α* (Figure 4(d)). This increase was abolished by nimodipine, a calcium channel blocker, and EGTA, a calcium chelator (Figure 4(d)). The data suggest that cytopiloyne activates PKC*α* through an increase of its activators, calcium and DAG.

### 3.5. Cytopiloyne Increases Insulin Secretion and Transcription via PKC*α*

**Figure 5:**
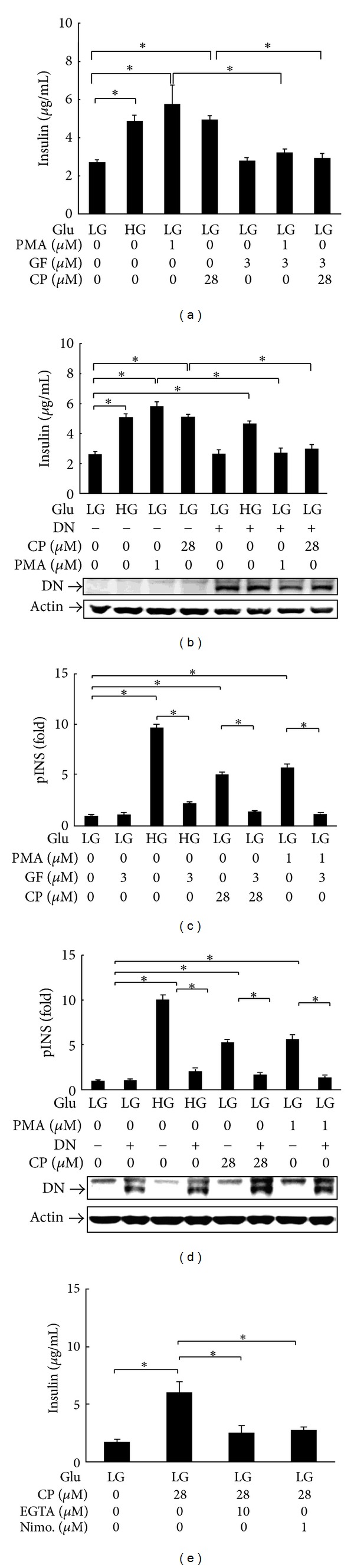
Cytopiloyne-mediated insulin secretion and expression are abolished by a dominant-negative mutant and a PKC*α* inhibitor. (a) RIN-m5F cells were grown in medium with 16.7 mM glucose (HG) or 3.3 mM glucose (LG) in the presence of PMA (1 *μ*M), GF109203X (GF, 3 *μ*M), and cytopiloyne (CP, 28 *μ*M). The insulin level in the supernatants was determined using an ELISA kit. (b) RIN-m5F cells were transfected with 5 *μ*g of pHACE-PKC*α* DN (+) or pcDNA3 (−) plasmid and grown in medium supplemented with 16.7 mM (HG) or 3.3 mM glucose (LG) in the presence of PMA and cytopiloyne. The insulin level was determined as described in (a). The expression level of dominant-negative HA-tagged PKC*α* (DN) and an internal control, actin, in the transfected cells was determined by Western blot using anti-HA and anti-actin antibodies. (c) RIN-m5F cells were transfected with phINS-Luc and pRL-TK plasmids. The cells were grown in medium with 16.7 mM (HG) or 3.3 mM glucose (LG) in the absence and presence of PMA, GF109203X, and cytopiloyne. The activity of the insulin promoter (pINS) in fold was measured using dual luciferase assays. (d) RIN-m5F cells were transfected with phINS-Luc and pRL-TK plus 5 *μ*g of pHACE-PKC*α* DN (+) or pcDNA3 (−) plasmids. The cells were grown in medium with 16.7 mM (HG) or 3.3 mM glucose (LG) in the absence or presence of PMA and cytopiloyne. Insulin promoter activity expressed as fold change relative to vehicle-treated control was measured using dual luciferase assays. The expression level of dominant-negative HA-tagged PKC*α* (DN) and actin in the transfected cells was determined using Western blot and anti-HA and anti-actin antibodies. (e) RIN-m5F cells were grown in medium with 3.3 mM glucose (LG) and/or cytopiloyne (CP, 28 *μ*M) in the presence of EGTA (10 *μ*M) or nimodipine (Nimo, 1 *μ*M). The insulin level in the supernatants was determined.

Next, we tested whether PKC*α* regulated cytopiloyne-mediated insulin secretion in *β* cells. We found that cytopiloyne stimulated insulin secretion in *β* cells, similar to what was observed in positive controls, 16.7 mM glucose and PMA (Figure 5(a)). By contrast, GF109203X, a PKC inhibitor, inhibited cytopiloyne- and PMA-induced insulin secretion (Figure 5(a)). Accordingly, overexpression of a dominant-negative mutant of PKC*α* decreased cytopiloyne- and PMA-mediated insulin secretion (Figure 5(b)). On the contrary, overexpression of this mutant only slightly, if at all, inhibited glucose-mediated insulin secretion (Figure 5(b)). These data showed that cytopiloyne increased insulin secretion in *β* cells in a PKC*α*-dependent manner. We also tested the involvement of PKC*α* in insulin transcription in *β* cells. We found that like 16.7 mM glucose and PMA, cytopiloyne stimulated insulin transcription in *β* cells (Figure 5(c)). GF109203X completely abolished cytopiloyne-, glucose-, and PMA-mediated insulin transcription (Figure 5(c)). Similarly, overexpression of a dominant-negative PKC*α* significantly inhibited insulin transcription (Figure 5(d)). These data revealed that cytopiloyne increased insulin transcription in *β* cells via PKC*α*. Furthermore, we tested whether calcium mobilization affected cytopiloyne-mediated insulin secretion. We found that calcium intervention inhibited the insulin secretion by cytopiloyne in RIN-m5F cells (Figure 5(e)) and rat *β* pancreatic islets (see the species list in the Supplementary Material of Figure  4 available online at http://dx.doi.org/10.1155/2013/685642). 

**Figure 6:**
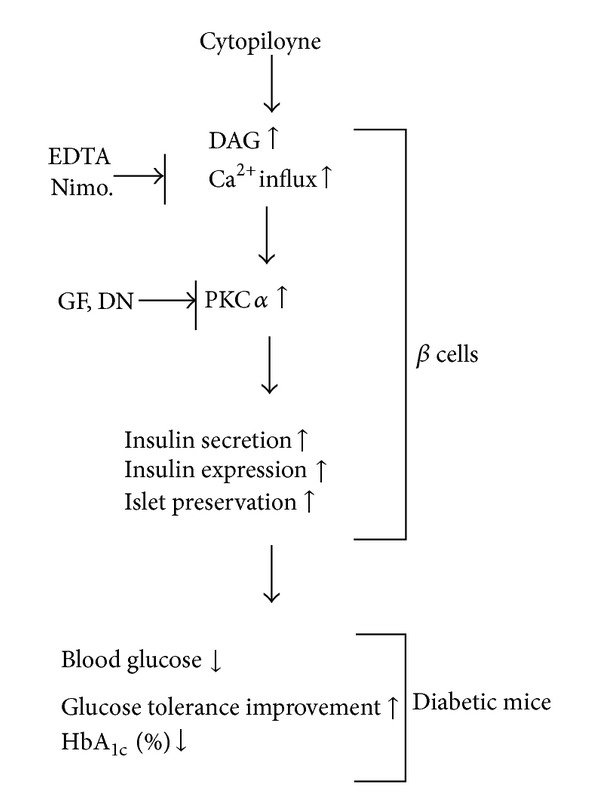
Schematic diagram of the likely mechanism by which cytopiloyne treats T2D in diabetic mouse models. Cytopiloyne shows anti-diabetic effects in diabetic mice, as evidenced by a reduction in the levels of blood sugar and glycosylated HbA_1c_, improvement of glucose tolerance, and its regulation of *β*-cell functions (e.g., insulin secretion, insulin expression, and pancreatic islet protection). The regulation of insulin secretion/expression in *β* cells by cytopiloyne involves PKC*α* and its activators, calcium, and DAG.

In summary, our mechanistic data suggest that cytopiloyne enhances insulin secretion and expression in *β* cells via the regulation of PKC*α* by calcium and DAG. The increase of insulin production in *β* cells and islet protection is associated with the therapy of cytopiloyne for T2D (Figure 6).

## 4. Discussion and Conclusions

Plants provide a promising source of anti-diabetic medicines. Cytopiloyne, a plant polyacetylene, represents a new class of anti-diabetic chemotherapeutics. This study not only demonstrates the anti-diabetic efficacy of cytopiloyne, but also reveals the mechanism of the anti-diabetic actions of cytopiloyne in cell and mouse models. 

Cytopiloyne has several unique benefits over current secretagogues in the market, including enhancement of insulin expression and maintenance of islet architecture. First, nutrients such as glucose can stimulate insulin biosynthesis at transcriptional and translational levels and the secretion of insulin in *β* cells [1, 36]. However, current secretagogues for diabetes can stimulate insulin secretion but not insulin biosynthesis. Unexpectedly, cytopiloyne can increase the level of insulin mRNA and protein, and it may be functionally superior to sulfonylureas (Figure 3). Second, cytopiloyne improved islet protection and resulted in a higher survival rate of db/db mice than glimepiride or vehicle control (Figure 1(F) and see the species list in the Supplementary Material of Table  1 available online at http://dx.doi.org/10.1155/2013/685642). Similarly, cytopiloyne was reported to maintain pancreatic islet architecture in NOD mice, a type 1 diabetes model [18]. Whether cytopiloyne employs the same mechanism in prevention of *β* cell death in both types of diabetes needs to be further investigated.

Cytopiloyne has other advantages over glimepiride. First, cytopiloyne is a relatively potent anti-diabetic compound as compared to glimepiride. Cytopiloyne induced similar anti-diabetic effects as glimepiride at one-fifth of the dose and modestly better anti-diabetic effects at the same dose (Figure 1 and Tables 1 and 2). In comparison with low-potency insulin secretagogues, cytopiloyne may have other benefits such as increased efficacy or decreased toxicity. Second, cytopiloyne may have a different mechanism of action as that of glimepiride, based on the large differences in their chemical structures. In fact, cytopiloyne, but not glimepiride, is able to promote insulin transcription (Figure 3) and confer islet protection (Figure 1(F)). Cytopiloyne is structurally different from currently known secretagogues and thus may represent a new class of anti-diabetic agents. Thus, studying the anti-diabetic mechanism of cytopiloyne may elucidate novel pathways that participate in insulin synthesis/secretion and islet preservation and the development of anti-diabetic agents.

No drug is perfect, and cytopiloyne presents challenges like other therapies. Current secretagogues occasionally reduce blood sugar to a detrimental degree, a condition known as hypoglycemia. The development of blood glucose-dependent secretagogues would prevent this dangerous side effect. At a low dose (e.g., 3 *μ*M), cytopiloyne stimulates insulin secretion in pancreatic islets in a glucose-dependent manner (see the species list in the Supplementary Material of Figure  3 available online at http://dx.doi.org/10.1155/2013/685642). However, a high dose of cytopiloyne can still stimulate insulin secretion to some extent even in the absence of glucose (Figure 2(a)). The data show that cytopiloyne-mediated insulin secretion is partially glucose dependent. Thus, at a high dose, cytopiloyne may pose a similar potential risk for hypoglycemia as sulfonylureas, particularly in patients with low blood glucose levels. However, this problem may be alleviated by decreased dosage or use in combination with a sensitizer such as metformin, which has no hypoglycemic effect. Additionally, it should be noted that insulin secretagogues have a low clinical incidence of hypoglycemia, because patients with T2D usually have higher insulin resistance than healthy subjects.

Cytopiloyne contains a glucose moiety and therefore it is conceivable that it acts at glucose receptors to mediate insulin expression and secretion. However, our data argue against this possibility. At 28 *μ*M, the concentration of glucose in cytopiloyne is about 600 times lower than the glucose in our experiments (16.7 mM). However, upregulation of insulin transcription by cytopiloyne at the same concentration is 40% (not 0.17%) of the up-regulation by 16.7 mM glucose (Figures 3(a) and 3(b)). Notably, 28 *μ*M cytopiloyne was less effective in inducing insulin mRNA production but more effective in increasing insulin protein levels than 16.7 mM glucose in islets (Figure 3). This discrepancy may be due to differences in the regulation of insulin at the mRNA and protein levels by cytopiloyne. Indeed, glucose and GLP-1 strongly stimulate insulin translation but only modestly stimulate insulin transcription in *β* cells [1, 37].

 DAG and calcium are common secondary messengers in *β* cells [31–35]. Both messengers activate PKC*α*. PKC*α* has been found to be involved in insulin secretion mediated by PMA and glucose [31–35] although the authors of one study excluded the participation of PKC*α* in glucose-mediated insulin secretion [31–35]. Consistent with the literature, our study shows that PMA activates PKC*α* in *β* cells to a greater extent than glucose (Figure 4(c)). In this study, cytopiloyne dose dependently activated PKC*α* (Figures 4(c) and 4(d)). PKC*α* activation of cytopiloyne was dependent on calcium (Figure 4(d)) and probably also DAG. Furthermore, interference with the PKC inhibitor, GF109203X, and the dominant-negative mutant of PKC*α* inhibited cytopiloyne-mediated insulin secretion/expression in *β* cells (Figures 5(a)–5(d)). The insulin secretion/expression of cytopiloyne is calcium dependent (Figure 5(e)). Therefore, our data suggest that cytopiloyne increases insulin production via PKC*α* activation that involves secondary messengers (Figure 6).

In conclusion, we showed that cytopiloyne suppressed the progression of T2D in db/db mice as evidenced by the decrease in the levels of blood glucose and HbA_1c_, glucose tolerance, and islet atrophy in db/db mice. Insulin release/expression and protection of *β* cells contributed to this suppression. We also showed that cytopiloyne up-regulated insulin release/expression that involved PKC*α* activation and its activators, calcium and likely DAG. This study reveals the anti-diabetic mode of action of cytopiloyne and suggests its use as a new anti-diabetic agent.

## Supplementary Material

Description of Figure 1: Effect of GF109203X on PKC*α* phosphorylation in beta cells.
Description of Table 1: Effect of cytopiloyne on Survival rate of diabetic mice.
Description of Figure 2: Prophylactic effect of cytopiloyne on diabetes in db/db mice.
Description of Figure 3: Effect of cytopiloyne on insulin release in rat islet cells in the absence and presence of glucose.
Description of Figure 4: Regulation of ion channels by cytopiloyne.

## Figures and Tables

**Table 1:** Blood glucose levels following a single oral dose of cytopiloyne in fed db/db mice. Diabetic db/db mice aged 6 to 8 weeks, with free access to food, were grouped and tube-fed with vehicle, glimepiride (GLM) at 2.5 mg/kg/day, and cytopiloyne (CP) at 0.1, 0.5, and 2.5 mg/kg/day. A half-hour after tube feeding was set as time 0. Blood samples were collected from the mice at the indicated time intervals (0, 1, 2, and 4 h). The blood glucose levels were determined using a glucometer. The number of mice (*n*) tested is indicated in parentheses in the first column.

Treatment	Blood glucose level (mg/dL)			
	0	1	2	4 (h)
Vehicle (*n* = 8)	365 ± 9	309 ± 4	281 ± 9	240 ± 11
GLM 2.5 mg/kg (*n* = 5)	378 ± 9	237 ± 16*	178 ± 20*	187 ± 6*
CP 0.1 mg/kg (*n* = 7)	373 ± 6	267 ± 12	204 ± 17*	158 ± 18*
CP 0.5 mg/kg (*n* = 7)	369 ± 27	214 ± 15*	163 ± 8*	132 ± 11*
CP 2.5 mg/kg (*n* = 7)	365 ± 9	207 ± 11*	147 ± 12*	129 ± 11*

*P < 0.05 as determined by ANOVA.

**Table 2:** Blood insulin levels following a single oral dose of cytopiloyne in fed db/db mice. Diabetic db/db mice aged 6 to 8 weeks received the same treatment as those in Table 1. Blood samples at the indicated time interval (0, 0.5, 1, 2, and 4 h) were collected from the mice and the insulin levels in each blood sample were determined using ELISA kits. The number of mice (*n*) is indicated in parentheses in the first column.

Treatment	Blood insulin level (*μ*g/L)				
	0	0.5	1	2	4 (h)
Vehicle (*n* = 8)	13.8 ± 1.9	11.5 ± 1.9	10.3 ± 2.4	8.3 ± 0.7	8.6 ± 1.9
GLM 2.5 mg/kg (*n* = 6)	13.9 ± 1.2	19.3 ± 4.2	26.3 ± 3.3*	15.9 ± 1.7*	11.8 ± 2.1*
CP 0.1 mg/kg (*n* = 5)	17.2 ± 4.7	21.0 ± 1.3*	15.5 ± 0.6*	14.1 ± 0.5	9.7 ± 1.3
CP 0.5 mg/kg (*n* = 5)	15.7 ± 1.9	22.6 ± 1.9*	16.0 ± 2.4*	12.8 ± 0.7*	12.2 ± 1.9
CP 2.5 mg/kg (*n* = 5)	12.5 ± 1.2	25.5 ± 1.2*	18.9 ± 3.3*	17.6 ± 1.7*	8.9 ± 2.1

*P < 0.05 as determined by ANOVA.

## Abbreviations

BW: Body weight
DAG: Diacylglycerol
DMSO: Dimethyl sulfoxide
FACS: Fluorescence-activated cell sorting
GLP-1: Glucagon-like peptide-1
HbA_1c_: Hemoglobin A_1c_
IPGTT: Intraperitoneal glucose tolerance test
IP3: Phosphatidylinositol triphosphate
KRB: Krebs-Ringer bicarbonate
PBS: Phosphate-buffered saline
PKA: Protein kinase A
PKC: Protein kinase C
PMA: Phorbol 12-myristate 13-acetate
STZ: Streptozocin
T2D: Type 2 diabetes.
