# Supplementary material for: Antidiabetic Effect and Mode of Action of Cytopiloyne
Source: Evid Based Complement Alternat Med. 2013 Mar 13;2013:685642. doi: 10.1155/2013/685642 (PMC3610345; doi:10.1155/2013/685642)

Supplemental data

Anti-diabetic effect and mode of action of cytopiloyne

Cicero Lee-Tian Chang[1], Hsien-Yueh Liu[1], Tien-Fen Kuo[2], Yi-Jou Hsu[2], Ming-Yi Shen[3], Chien-Yuan Pan[4], and Wen-Chin Yang[2,4,5*]

[1]Department of Veterinary Medicine, National Chung Hsing University, Taiwan;
[2]Agricultural Biotechnology Research Center, Academia Sinica, Taiwan; [3]Research Center, China Medical University and Hospital; Graduate Institute of Clinical Medical Sciences, China Medical University, Taiwan;
[4]Institute of Zoology, National Taiwan University, Taiwan;
[5]Department of Life Science, National Chung Hsing University, Taiwan.

Supplemental Table 1. Survival rate of db/db mice during long-term treatments.

	Survival rate (%)			
Treatment	6	10	20	22 (week)
Vehicle (n=4)	100	100	75	75
GLM 2.5 mg/kg (n=4)	100	100	100	75
CP 0.5 mg/kg (n=7)	100	100	100	100
CP 2.5 mg/kg (n=7)	100	100	100	100

Each group of db/db mice aged at 6 to 8 weeks were tube-fed with vehicle, cytopiloyne (0.5 or 2.5 mg/kg/day) or glimepiride (2.5 mg/kg/day) once a day. Survival rate was monitored for the indicated time. The number of mice used is indicated in parentheses in the first column.

Supplemental Figure Legends

Supplemental Figure 1. GF109203X can inhibit PKCα phosphorylation in β cells. RIN-m5F cells were pre-treated with GF109203X (GF), a pharmacological agent, for 24 h and stimulated with PMA for 5 min. The cells were lysed and underwent Western blot with anti-phospho-PKCα and anti-actin antibodies.

Supplemental Figure 2. Prophylactic effect of cytopiloyne in db/db mice. (A) Two groups of diabetes-free db/db mice aged 4 weeks were daily tube-fed with vehicle (1 μl DMSO per 1 ml of PBS) or cytopiloyne (CP, 0.5 mg/kg), for 4 weeks. Postprandial blood glucose levels in these mice were measured. (B) Blood insulin levels from the above mice (A) were measured. Results from 3 independent experiments are expressed as mean ± SEM, and P (*) < 0.05 was considered to be statistically significant. The mouse number (n) is indicated in parentheses.

Supplemental Figure 3. Cytopiloyne dose-dependently enhances insulin release in the medium containing different doses of glucose. Rat pancreatic islets were incubated with KRB buffer containing vehicle, glimepiride (GLM, 10 μM) or cytopiloyne at 1.5, 3, 7, 14, 28 and 42 μM in the presence of 16.7 mM glucose (HG, upper row) or 3.3 mM glucose (LG, middle row). Similarly, rat pancreatic islets were incubated with glucose-free KRB buffer containing vehicle or cytopiloyne at 1.5, 3, 7, 14, 28 and 42 μM (lower row). The insulin levels were determined using an insulin ELISA kit. The data are presented as mean ± SEM of 3 independent experiments. All the results from 3 independent experiments are expressed as mean ± SEM, and P (*) < 0.05 was considered to be statistically significant.

Supplemental Figure 4. Cytopiloyne-mediated insulin release depends on calcium and potassium channels. Rat pancreatic islets were incubated with KRB buffer containing vehicle or cytopiloyne (CP, 42 μM) in the presence of calcium channel inhibitors (10 μM EGTA and 1 μM nimodipine (Nimo)) and potassium channel opener (100 μM diazoxide (Diaz)) for 30 min. Insulin levels in KRB buffer were determined. All the data are presented as mean ± SEM of 3 independent experiments and P (*) < 0.05 was considered to be statistically significant.

Supplemental Figure 1

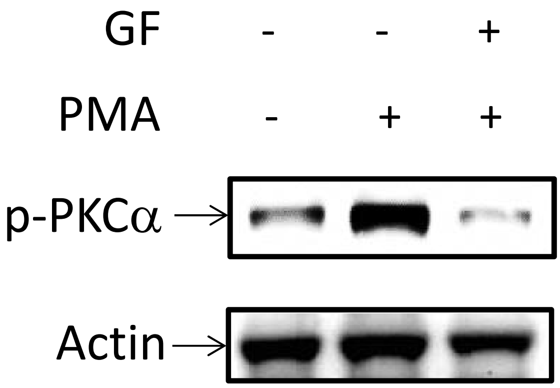

Supplemental Figure 2

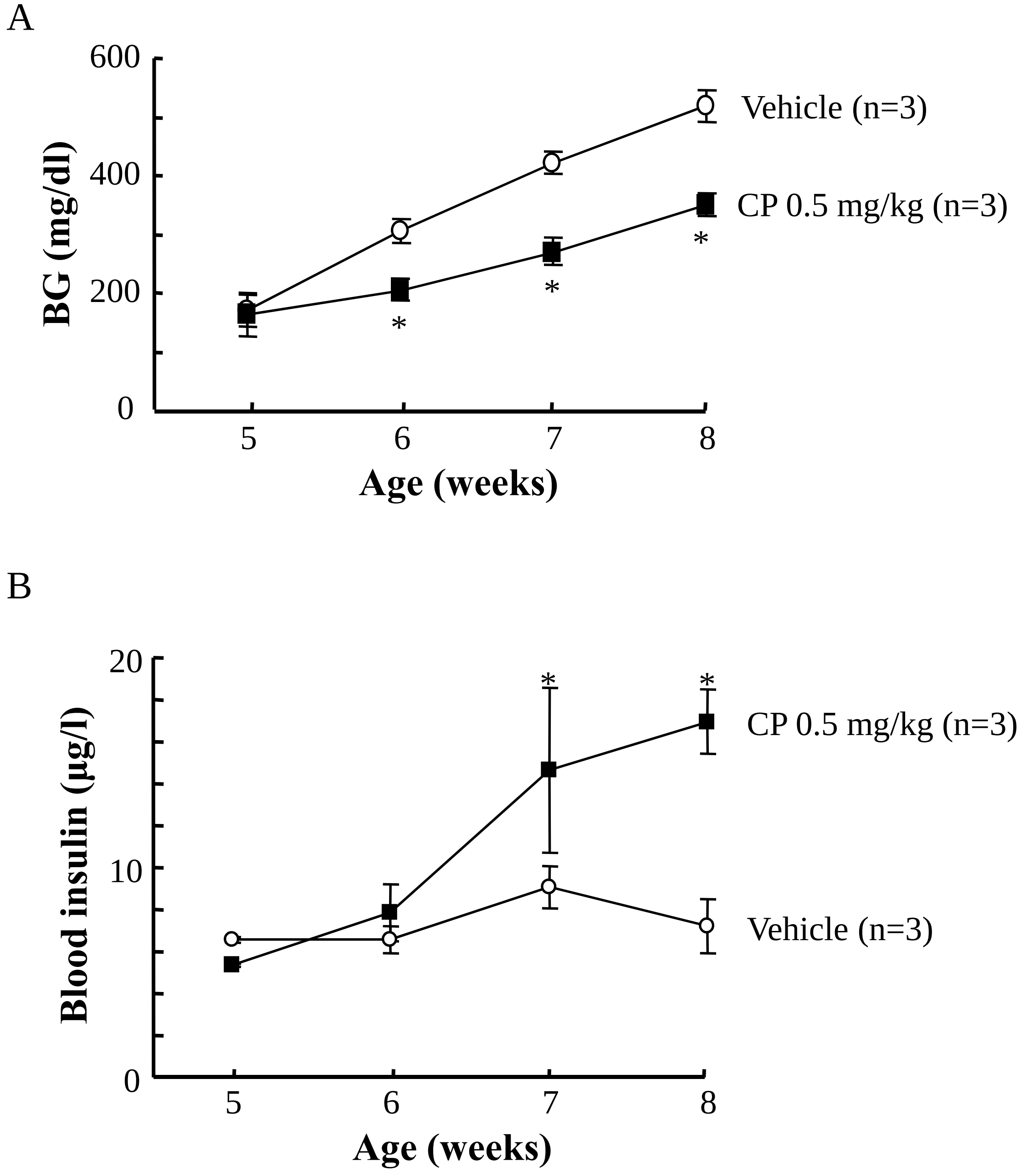

Supplemental Figure3

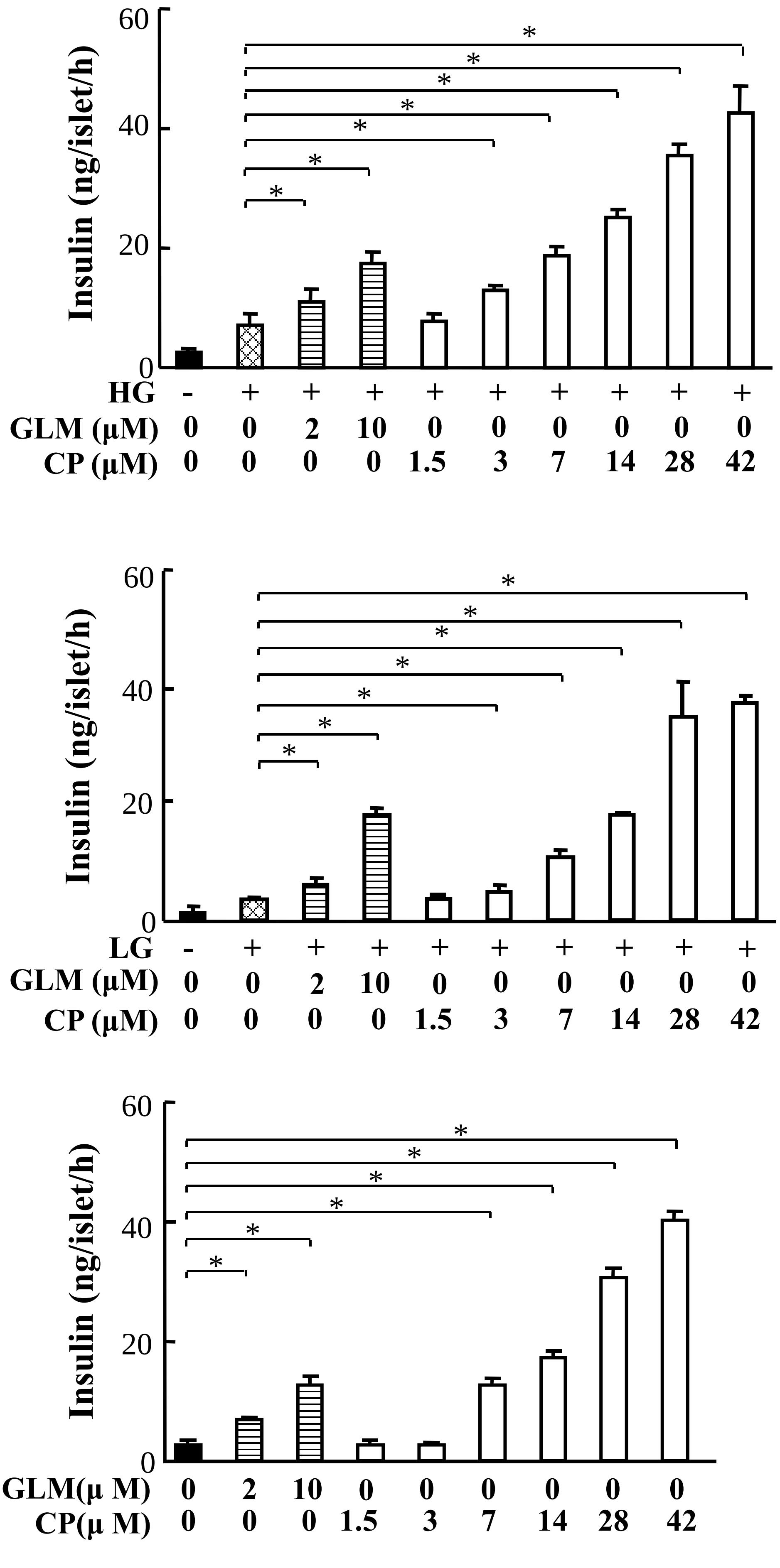

Supplemental Figure 4

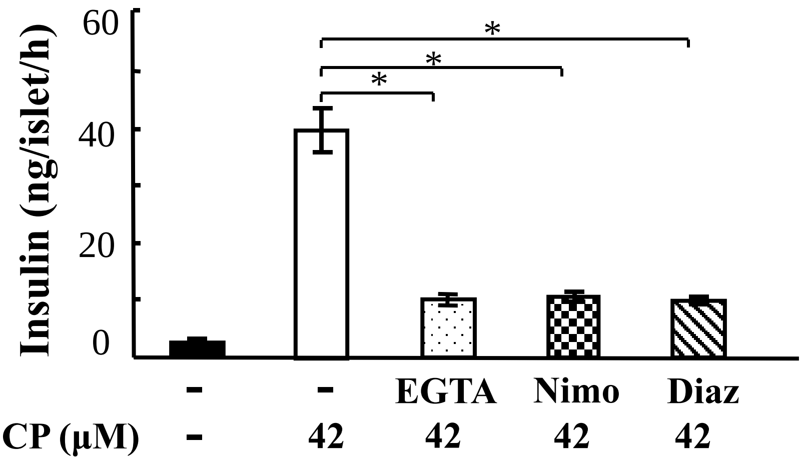

Supplement: Supplementary file 1 — Description of Figure 1: Effect of GF109203X on PKCα phosphorylation in beta cells. Description of Table 1: Effect of cytopiloyne on Survival rate of diabetic mice. Description of Figure 2: Prophylactic effect of cytopiloyne on diabetes in db/db mice. Description of Figure 3: Effect of cytopiloyne on insulin release in rat islet cells in the absence and presence of glucose. Description of Figure 4: Regulation of ion channels by cytopiloyne. [file 685642.f1.pdf]
